# Negative Feedback Does Not Reverse Observationally Acquired Binding and Retrieval Effects: A Failed Replication

**DOI:** 10.5334/joc.494

**Published:** 2026-03-27

**Authors:** Kira Franke, Klaus Rothermund, Bernhard Hommel, Carina G. Giesen

**Affiliations:** 1 Department of General Psychology II, Friedrich-Schiller University Jena, Jena, Germany; 2 Department of Psychology, Shandong Normal University, Jinan, Shandong Province, China; 3 Department of Psychology, Faculty of Health, HMU Health and Medical University Erfurt, Erfurt, Germany

**Keywords:** stimulus-response binding, event files, vicarious feedback, observational learning, episodic retrieval

## Abstract

In the present study, we ran a replication of the experiment by Giesen et al. (2017) who found reversed observationally acquired stimulus-response binding and retrieval (oSRBR) effects after negative feedback and standard oSRBR effects after positive feedback. This suggests that feedback was used to infer action goals from observed actions, implying that oSRBR effects represent propositional information. However, their findings stand in contrast to recent studies on the influence of affective consequences like feedback on SR bindings stemming from self-performed actions. These studies consistently demonstrate that SR binding and retrieval effects emerge independently of feedback. This raises the question whether the findings by Giesen et al. (2017) reflect an alpha error. In our replication, we found no evidence for a modulatory influence of feedback on oSRBR effects. A meta-analysis with the data from Giesen et al. (2017) and our experiment revealed no significant modulatory effect of vicarious feedback on retrieval of observationally acquired SR bindings. In concert with recent literature, this implies that feedback information was not used to infer action goals, providing no evidence that oSRBR effects represent propositional information.

Binding and retrieval are core processes of human action control. When a response, like a key press, is executed in close temporal proximity to a stimulus (e.g., a word), the mental representations of stimulus and response are temporarily bound into a stimulus-response (SR) binding or event file (Hommel et al., 2001; for overviews see Frings et al., 2020; 2024). When the stimulus is reencountered, the entire SR binding is retrieved from memory, and the response is reactivated. This impacts on performance: If the retrieved response has to be executed again in the current situation, responding is facilitated. This is typically reflected in faster reaction times and/or lower error rates compared to when the stimulus changes (i.e., when no retrieval takes place). In contrast, if the retrieved response is inappropriate, this typically interferes with performance, leading to slower reactions and/or higher error rates. Statistically, the presence of SR binding and retrieval effects is reflected in a two-way interaction of stimulus relation and response relation.

Several studies demonstrated that SR bindings can also be formed when the response is only observed in another person. These *observationally acquired* SR bindings can then be retrieved and are used to guide one’s own performance in the same way as SR bindings stemming from self-performed actions (Franke, Keller, et al., 2025; Franke, Rothermund, et al., 2025; Giesen et al., 2014; 2017; 2018; 2021; Giesen & Rothermund, 2022). In the lab, observationally acquired SR binding and retrieval (oSRBR) effects are typically investigated using a dyadic color categorization task (Giesen et al., 2014) that follows a prime-probe structure. In the prime trial, one participant (the prime actor) categorizes a word according to its font color (usually red or green) by pressing a buzzer in the corresponding color. At the same time, the other participant sees the same word in white font and simply observes their co-actor’s response. This should be sufficient for the emergence of an observationally acquired SR binding. In the subsequent probe trial participants switch roles: The former prime observer becomes the probe actor and has to perform the categorization response. Prime words either repeat or change. To-be-executed probe responses are either identical to or different from observed prime responses. In this constellation, probe performance typically reflects the standard pattern indicative of SR binding and retrieval. Crucially however, such oSRBR effects only occur if certain conditions are met, for example if the co-actor is socially relevant to the probe actor. Social relevance can, for example, stem from situational interdependence like being instructed to cooperate or compete for an extra reward (Giesen et al., 2014) or chronic interdependence when the co-actor is also the participant’s romantic partner (Giesen et al., 2018). Interestingly, the modulating factors that determine whether an observationally acquired SR binding will be used to guide one’s own actions closely resemble known moderators of social learning (Bandura, 1986). Because of this, it has been proposed that SR binding and retrieval may be the basic cognitive processes that underly social learning phenomena (Giesen, 2024).

Another important factor that influences social learning is vicarious feedback: Bandura (1965) found that children were less likely to imitate observed actions if the observed model was punished for those actions compared to conditions in which the model was either rewarded or received no feedback. Interestingly, Giesen et al. (2017) demonstrated that oSRBR effects were also affected by vicarious feedback. In their study, they expanded the standard dyadic color categorization task by presenting feedback to both participants after each response, meaning that the observer received *vicarious feedback* for their partner’s response. When observed prime responses were followed by positive feedback, standard oSRBR effects emerged (in error data). However, after negative feedback, the pattern was *reversed*: Error rates were lower when the to-be-executed probe response was different from the previously observed prime response in stimulus repetition trials compared to stimulus changes. Conversely, error rates were higher when prime and probe responses were identical in stimulus repetition vs. change trials. This pattern of results suggests that after negative feedback, the alternative instead of the observed response was integrated into the event file. Giesen et al. (2017) explained this with the overlearned tendency to interpret negative feedback as error feedback, that is, feedback that was triggered by a wrong response. In such a situation, prime observers possibly infered that the other one of the two available response options would have been correct. According to this explanation, observers actively inferred action goals based on the available information (i.e., observed response plus negative feedback), leading to the binding of the inferred correct response rather than the observed response.

If such a logical inference can indeed influence which response becomes bound, this has important implications for the nature of observationally acquired SR bindings. It would suggest that observationally acquired SR bindings may not represent transient episodic linkages between co-occurring stimuli and the observed responses in an unqualified manner. Instead, they may represent propositions that contain information on the (inferred) relation between co-occurring events and imply a truth value (e.g., De Houwer, 2009).

The idea that SR bindings may represent goal-based information rather than transient episodic linkages of co-occurring stimuli and responses primarily receives support from research on action slips, that is, situations in which one fails to act as intended. It has repeatedly been demonstrated that after an error is committed, the correct response instead of the executed erroneous response is retrieved when reencountering the same stimulus, a mechanism that has been denoted as “goal-based binding” (Foerster et al., 2023; Foerster et al., 2022; Foerster et al., 2021; Parmar et al., 2022). This has been interpreted as evidence that binding is a highly adaptive mechanism that promotes the success of future actions by selectively coding the features of the intended (correct) action. Following this notion, it is tempting to assume that external signals that imply that a response was incorrect, like negative vicarious feedback, may trigger similar processes and lead to binding of the response that is assumed to be correct (i.e., the inferred action goal).

However, the findings from previous studies on modulating influences of external feedback on SR bindings offer little insight to this unresolved issue. Whereas Colzato et al. (2007) and Waszak and Pholulamdeth (2009) found modulations of SR bindings by positive versus negative effects following responses, several recent studies (Martini et al., 2025; Mocke et al., 2025; Parmar & Rothermund, 2024; Schöpper et al., in press) document that SR binding was not modulated by affective consequences in a series of several highly powered experiments. Affective consequences were implemented in the form of either performance-dependent or performance-independent feedback after each response. In all experiments, significant SR binding and retrieval effects emerged regardless of whether the response had been followed by positive, neutral, or negative feedback. Therefore, these studies concluded that SR binding and retrieval processes are highly automatic mechanisms that are impenetrable by affective consequences such as feedback.

At first glance, these findings stand in contrast to those of Giesen et al. (2017). However, there are important differences between the studies that may reconcile these differing findings. Most importantly, the negative feedback in the studies by Parmar and Rothermund (2024), as well as in the studies by Martini et al. (2025), Mocke et al. (2025), and Schöpper et al. (in press) did not signal an error. Instead, it was either random or, in the case of performance-dependent feedback, indicated slow but correct responding. Thus, if the explanation for the modulation of oSRBR effects by vicarious feedback in terms of propositional goal inference by Giesen et al. (2017) is indeed correct, one would not expect feedback to modulate SR binding and retrieval in these experiments. On the other hand, the modulation of oSRBR effects by vicarious feedback was limited to error rates, and did not emerge for response latencies. Therefore, it needs to be considered that the findings by Giesen et al. (2017) may merely reflect a chance finding. That is because apart from their study, evidence for goal-based binding has only been found in the context of action slips. Committing an error yourself, however, is different from receiving negative feedback for an observed action and then concluding that the observed action must have been incorrect and that another action should have been executed instead.

To test these two competing explanations, we decided to replicate the study by Giesen et al. (2017). If observationally acquired SR bindings indeed contain propositional goal inferences, then we should be able to replicate their findings. In this case, we would expect a three-way interaction of stimulus relation, response relation, and vicarious prime feedback, with standard oSRBR effects after positive feedback and reversed oSRBR effects after negative feedback. However, if the findings of Giesen et al. (2017) reflect an alpha error and feedback does not modulate oSRBR effects, there should be no three-way interaction. Instead, standard oSRBR effects should emerge after both positive and negative feedback.

## Method

### Transparency and openness

Prior to data collection the exact method, design, data preparation, and planned analyses were preregistered online at the Open Science Framework (OSF, https://osf.io/xcb4n). All data, analysis scripts, and experimental files are available on OSF (https://osf.io/rntf9). The experiment was in accordance with the Ethical standards of the Institute of Psychology of the University of Jena and the Declaration of Helsinki.

### Participants

In the study by Giesen et al. (2017) an effect size of *d*_z_ = 0.35 was observed. According to an a-priori power analysis with G*Power 3.1.9.7 (Faul et al., 2007), a sample size of n = 72 is necessary to show an effect of *d*_z_ = 0.35 with a power (1–ß) = 0.90 in a one-tailed *t*-Test for dependent samples (contrasting oSRBR effects after positive and negative vicarious feedback) with α = 0.05.

Overall, 74 students of the university of Jena took part in the experiment. They received 9€ as a reward for completing the entire experiment and chocolate as an extra reward. Three participants were excluded due to excessive errors rates (>25%) in either the color categorization task or in the memory test. Another three participants were excluded because they were not native German speakers. Thus, data of 68 participants (34 male, 34 female; *M*_age_ = 21, *SD*_age_ = 2.8) were analyzed.

### Design

The experiment comprised a 2×2×2 within-subject design with the factors stimulus relation, response relation, and vicarious prime feedback. Stimulus relation was manipulated by either repeating or changing the word stimulus from prime to probe. Response relation was manipulated by having probe actors either perform responses that were identical to (response repetition; e.g., red-red) or different from (response change; e.g., green-red) observed prime responses. Each prime response was followed by either positive (for fast and correct responses) or negative feedback (for incorrect or correct but slow responses) that was audio-visually presented to both participants. Authentic positive prime feedback was given after very fast correct responses (i.e., RTs within the first quartile of the individual RT distribution sampled from the 20 preceding trials), whereas authentic negative prime feedback was given after incorrect or very slow correct responses (i.e., RTs within the fourth quartile of the individual RT distribution). This was done to ensure face validity of both feedback levels, as participants typically have accurate insight whether a response was (a) correct or incorrect or (b) whether a given response was extremely fast or slow. To ensure roughly equal rates of both feedback levels, feedback valence was determined randomly (50% positive, 50% negative) for RTs in the middle range (second or third quartile of the individual RT distribution). This manipulation resulted in rates of 48% positive (52% negative) prime feedback and can thus be considered successful.

Error rates (ERR) and reaction times (RT) in probe trials served as our main dependent variables of interest.^1^

### Apparatus and stimuli

**Figure 1:**
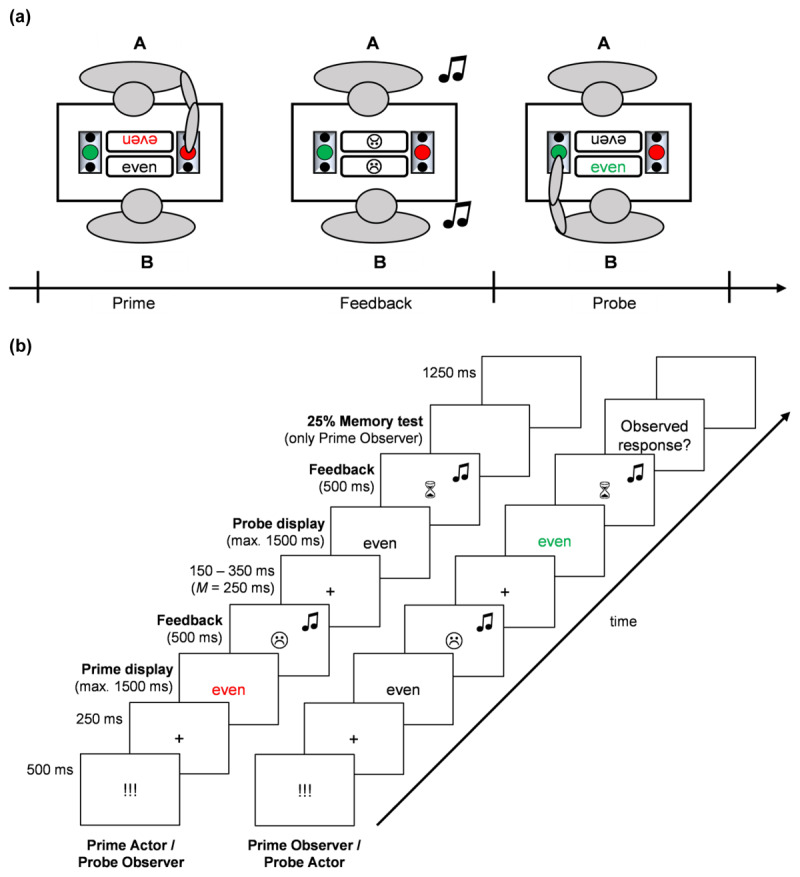
Schematic illustration of **(a)** the experimental setup and **(b)** and a sample trial sequence from the perspective of each co-actor. *Note*. Stimuli are not drawn to scale. For illustrative purposes, foreground and background colors are inverted.

The original experiment by Giesen et al. (2017) was updated from E-Prime 2.0. to E-Prime 3.0. to be compatible with the more modern operating system that was used for the data collection. Participant pairs were placed opposite of each other, each in front of a 21-inch screen. The screens were positioned to prevent eye contact between participants (Figure 1a). However, they could still see the two response pads that were used to collect responses, and thus the other participant’s responses, with their peripheral vision. One of these response pads had a green and the other one a red push button in the middle. Both had two black rest state-keys in front of and behind the push button. During the color categorization task, participants continuously pressed the two rest-state keys on their side with their right and left hand and only released those keys to respond by hitting the red or the green button. The response pads were connected to the computer via a parallel port.

We used 25 neutral mono- or disyllabic German adjectives as stimuli (e.g., glatt, eben, kurz; in English: smooth, even, short). These adjectives were presented centrally on each participant’s screen in Times New Roman font (font size: 16 pt). They were either presented in white (RGB: 255, 255, 255), green (RGB: 20, 156, 23), or red (RGB: 255, 0, 0) color. For auditory feedback, we used two different computer-generated sounds, presented to participants via headphones. For both positive and negative feedback, sounds started at 440 Hz. Positive feedback sounds increased in steps of 20 Hz (each step lasting for 15 ms) until a frequency of 840 Hz was reached. For negative feedback, sounds decreased at the same rate until a frequency of 40 Hz was reached. This resulted in sounds that are intrinsically positive (increasing frequencies) or negative (decreasing frequencies; cf. Rothermund, 2003). Total sound duration of the positive and the negative feedback sound was 300 ms.

### Procedure

First, demographic information (age, gender, study field) was collected, and participants gave their informed consent to participate; otherwise the study was terminated. Then written instructions for both participants were presented on each of their screens. Participants were informed that they would perform a color categorization task together with the other participant. This task followed a socially shared prime-probe design (Giesen et al., 2014; 2018) and was used to assess oSRBR effects. In both the prime and the probe trial the participants’ task was to categorize the font color of the presented word stimulus as fast and accurately as possible by pressing the corresponding (i.e., red/green) push button in the middle of the response pads. Word stimuli could appear with an equal likelihood of 50% in either green or red font. Further, participants were instructed that positive feedback indicated that a response was both correct and fast, while negative feedback could signal either an error or that a response was correct but too slow. However, feedback was only authentic for very fast or very slow RTs and incorrect responses (see Design for details).

In each prime or probe trial, only one participant (the actor in the respective trial) saw the word in green or red color and performed the color categorization response. The other participant (the observer) saw the same word in white font and had to observe the actor’s response. Participants responded in strict alternation: During the first half of the experiment (prime-probe sequences 1–180), participant A was the prime actor/probe observer, meaning that participant A always gave the color categorization response in the prime trial and observed participant B’s response in the probe trial. Participant B was the prime observer/probe actor. For the second half of the experiment (prime-probe sequences 181–360) these roles were switched: now participant A was prime observer/probe actor and participant B prime actor/probe observer.

Each prime-probe sequence of the color categorization task (see Figure 1) began with a ready signal (“!!!”) that was presented centrally on both participants’ screens in white font against a black background for 500 ms, followed by a fixation cross (250 ms). Next, the prime word was presented until the prime actor responded or until 1500 ms had passed (i.e., the prime actor failed to respond in time). The word was shown in red or green font to the prime actor and in white font to the prime observer. Then, feedback for the prime actor’s response was shown to both participants for 500 ms. In case of positive feedback, participants saw a smiley face and heard the positive feedback sound. Negative feedback included a schematic grumpy face and the negative feedback sound. After that, the probe trial began with the presentation of another fixation cross for a variable duration of 150 to 350 ms (*M* = 250 ms). Then the probe word (printed in green or red for the probe actor and in white for the probe observer) appeared on the screen until the probe actor responded or until a maximum of 1500 ms elapsed. Both participants also received feedback for the probe actor’s response (duration: 500 ms). This probe feedback was determined by the same rules as the prime feedback. However, probe feedback was not of theoretical interest and was only used to make conditions comparable for both prime and probe actors.

To ensure that prime observers attended to prime responses, 25% of all probe trials were followed by a memory test. Prime observers were asked to repeat the prime actor’s response they had just observed by pressing the corresponding push-button (until response). Each prime-probe sequence ended with a blank black screen (1250 ms).

Before the start of the first block of the main experiment, participants completed a practice block consisting of 18 prime-probe sequences. The practice block had to be repeated if more than 20% of the categorization responses were incorrect. If participants did not pass the practice block upon the third try the experiment was terminated. Additionally, there was a shorter practice block of 8 prime-probe sequences before the second block of the main experiment for participants to get accustomed to the role change. To increase the likelihood of the occurrence of oSRBR effects, the experiment adopted the “positive interdependency” manipulation that was first used by Giesen et al. (2014). Before the start of the main block, participants were instructed to work together and were told that they would only receive an extra reward (a chocolate bar) if they both performed well in terms of accuracy (i.e., less than 25% errors in the color categorization task and in the memory test). Participants then worked through two experimental blocks of 180 prime-probe sequences each. After every 60 prime-probe sequences, participants could take a short break. During these breaks, the interdependency manipulation was refreshed by reminding participants to work together to obtain the extra reward.

After completion of the color categorization task, participants were asked to fill out a questionnaire on how they experienced the experimental situation and their interaction partner. On 7-point Likert scales they rated the situation as cooperative (1) vs. competitive (7; one item) and comfortable vs. uncomfortable (averaged across three items: 1 = difficult/unpleasant/negative; 7 = easy/pleasant/positive; Cronbach’s α = .57). Further, they were asked how agreeable they perceived their interaction partner (averaged across four items: 1 = disagreeable/insecure/unfriendly/incompetent; 7 = agreeable/confident/friendly/competent; Cronbach’s α = .81). Finally, participants were debriefed and rewarded.

### Data preparation

Prior to all analyses, probe trials with errors in the memory test (5.89%, 1.96% of all trials) and RT outliers (5.39%), that is, trials with probe RTs faster than 300 ms or slower than 3 interquartile ranges above the 75^th^ percentile of the individual RT distribution (Tukey, 1977), were discarded. Deviating from our preregistration, we also excluded trials in which the wrong person responded (0.61%). For RT analysis, we excluded all trials with errors in the color categorization task (6.02%). Means for probe RTs and ERR for all conditions of the factorial design are provided in Table 1. Additionally, effect scores for oSRBR effects representing the size of the stimulus relation × response relation interaction were calculated for each participant (see Table 1 for computation).

**Table 1:** Probe performance M (SD) in the observational SR binding paradigm.


VICARIOUS PRIME FEEDBACK		% ERRORS		RT (ms)	
	
		RR	RC	RR	RC

Positive	Stimulus repetition (SR)	5.5 (8.6)	5.0 (6.6)	426 (61)	438 (65)

	Stimulus change (SC)	4.3 (5.6)	3.8 (6.2)	434 (65)	437 (64)

	Δ SC – SR	–1.2 [1.1]	–1.2 [1.0]	8* [2.7]	–1 [2.7]

	S × R interaction score	0.0 [1.6]		9* [3.8]	

Negative	SR	6.0 (6.3)	6.1 (7.5)	442 (65)	440 (67)

	SC	5.5 (6.7)	3.9 (6.4)	443 (68)	442 (67)

	Δ SC – SR	–0.5 [0.8]	–2.2* [0.9]	1 [2.9]	2 [2.7]

	S × R interaction score	1.7 [1.3]		–1 [4.1]	


*Note. RR* = response repetition, RC = response change. S × R interaction score = (Δ SC – SR)_RR_ – (Δ SC – SR)_RC_. Standard error of the mean in brackets. * *p* < .05. ** *p* < .01. *** *p* < .001. Asterisks denote that effects significantly differ from zero.

## Results

### Manipulation checks

A *t*-test against the scale midpoint (4) showed that participants perceived the experimental situation as rather cooperative, *M* = 3.68, |*t*|(67) = 1.74, *p* = .043, |*d*| = 0.21, implying that positive interdependence was induced successfully. Also, participants thought the situation was rather comfortable, *M* = 4.31, *SD* = 0.88, and that their interaction partner was agreeable, *M* = 5.57, *SD* = 1.01.

Also, errors rates in the memory test did not differ significantly between prime feedback levels, *t*(67) = 1.54, *p* = .129, *d* = 0.19. This indicates that participants attended and remembered observed responses equally well regardless of the vicarious feedback level (*M*_positive_ = 2.82%, *M*_negative_ = 3.92%). Thus, potential differences in retrieval of observationally acquired SR bindings cannot be explained by these factors.

### Preregistered analyses

#### Response times

**Figure 2:**
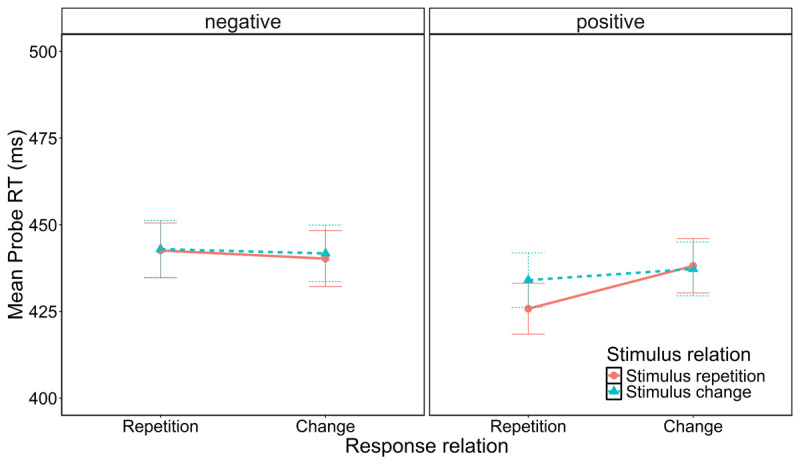
Probe performance (RT) as a function of stimulus relation, response relation, and vicarious prime feedback.

Probe RTs were entered into a 2 (stimulus relation: stimulus repetition vs. stimulus change) × 2 (response relation: response repetition vs. response change) × 2 (vicarious prime feedback: positive vs. negative) repeated measures ANOVA. The global ANOVA results can be found in Table 2. There was a significant main effect of prime feedback, showing that participants tended to respond faster when their interaction partner had previously received positive feedback in the prime (*M* = 434 ms) than when the partner had received negative feedback (*M* = 442 ms). Further, there was a significant interaction of response relation and prime feedback. No other effect was significant (*F* ≤ 2.69, *p* ≥ .106). Most importantly, neither the interaction of stimulus relation and response relation (*F* < 1), nor the three-way interaction of stimulus relation, response relation, and vicarious prime feedback were significant, *F*(1,67) = 3.32, *p* = 0.072, *η*_p_^2^ = 0.05, BF_01_ = 1.58. Thus, in RT data, vicarious prime feedback did not affect retrieval of observationally acquired SR bindings (see Figure 2).

**Table 2:** ANOVA results on probe actors’ mean error rates, and mean RTs.


	VARIABLES	*df*1	*df*2	*F*	*p*	*η*_P_^2^

Errors						

	Stimulus relation (S)	1	67	11.76**	.001	.15

	Response relation (R)	1	67	1.26	.266	.02

	Prime Feedback (F)	1	67	2.12	.150	.03

	S × R	1	67	0.60	.441	.01

	S × F	1	67	0.02	.889	<.01

	R × F	1	67	0.14	.714	<.01

	S × R × F	1	67	0.77	.382	.01

RT						

	S	1	67	2.69	.106	.04

	R	1	67	1.71	.196	.02

	F	1	67	20.00***	<.001	.23

	S × R	1	67	2.13	.149	.03

	S × F	1	67	1.13	.291	.02

	R × F	1	67	10.80**	.002	.14

	S × R × F	1	67	3.32	.073	.05


*Note*. * *p* < .05. ** *p* < .01. *** *p* < .001.

When testing effect scores against zero for each prime feedback condition individually, the *t*-tests revealed that effect scores differed significantly from zero after positive, S×R_positive_ = 9 ms, *t*(67) = 2.45, *p* = .017, *d* = 0.30, BF_01_ = 0.48, but not after negative prime feedback, S×R_negative_ = –1 ms, |*t|*(67) = 0.27, *p* = .788, |*d|* = 0.03, BF_01_ = 7.25. Descriptively, this suggests that observationally acquired SR bindings were retrieved after positive but not after negative feedback, although the difference in retrieval effects failed conventional levels of significance (test of three-way interaction).

#### Error rates

The same 2 × 2 × 2 ANOVA (see Table 2 for results) on mean error rates revealed a significant main effect of stimulus relation. Participants on average responded more accurately when words changed from prime to probe (*M* = 4.35%) than when they repeated (*M* = 5.63%). All other effects were not significant (all *F* ≤ 2.12, all *p* ≥ .150), including the two-way interaction (*F* < 1) of stimulus relation and response relation and the three-way interaction (*F* < 1, BF_01_ = 5.19). This indicated that there was no retrieval of observationally acquired SR bindings in error data and that oSRBR effects were also not influenced by vicarious prime feedback.

Further, interaction effect scores were tested against zero in a two-tailed *t*-test for each feedback condition. The *t*-test was neither significant in the positive, S×R_positive_ = 0.01, *t*(67) = 0.01, *p* = .993, *d* = 0.00, BF_01_ = 7.51, *M* = 0.01%, nor in the negative feedback condition, S×R_negative_ = 1.69, *t*(67) = 1.27, *p* = .209, *d* = 0.15, BF_01_ = 3.50, *M* = 1.69%, suggesting that oSRBR effects were absent in both conditions.

### Discussion

The present experiment was an exact replication of the study by Giesen et al. (2017). We conducted this experiment to test whether oSRBR effects can actually be modulated by vicarious feedback or if it is more likely that the results of Giesen et al. (2017) were a chance finding. First, we found a main effect for vicarious prime feedback in RTs, with responses being faster after positive feedback compared to negative feedback. This implies that participants processed and were affected by our feedback manipulation. Second, we found that significant standard oSRBR effects only occurred after positive feedback, while oSRBR effects were absent after negative feedback. However, this difference between feedback conditions was not significant. Therefore, overall, our results suggest that binding and retrieval by observation was not affected by vicarious prime feedback. This deviates from the findings of Giesen et al. (2017), as retrieval effects were not reversed after negative feedback, but absent.

Regarding our research question, our findings do not support the claim that SR bindings represent propositional goal inferences or that feedback has a modulatory influence on observational SR binding and retrieval in general. The size of oSRBR effects did not differ significantly between feedback conditions, and oSRBR effects were not reversed after negative feedback. Taken together, it therefore seems likely that the findings by Giesen et al. (2017) reflect an alpha error.

Our findings render it unlikely that SR bindings acquired by observation represent propositional goal inferences or that vicarious feedback modulates oSRBR effects at all. However, to rule out the possibility that we did not find an effect because of insufficient power, we conducted an exploratory meta-analysis on the combined data of our experiment and of the study by Giesen et al. (2017).

## Meta-analysis

**Figure 3:**
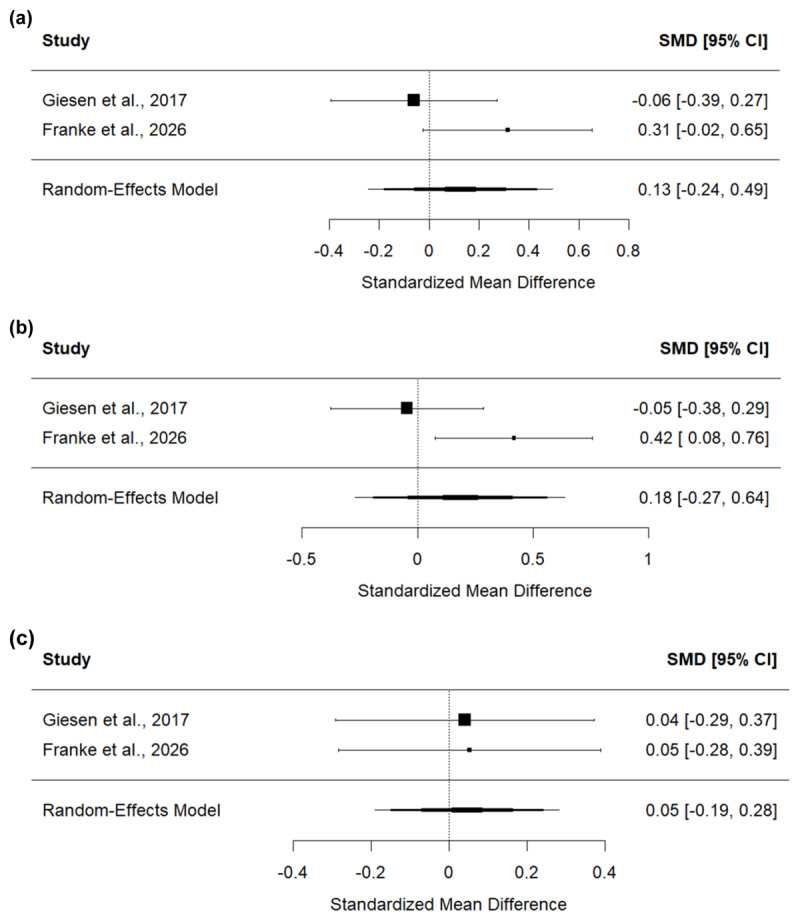
Forest plots for the RT meta-analyses on **(a)** the modulating effect of vicarious feedback on oSRBR effects and **(b)** the occurrence of oSRBR effects after positive and **(c)** negative feedback respectively.

**Figure 4:**
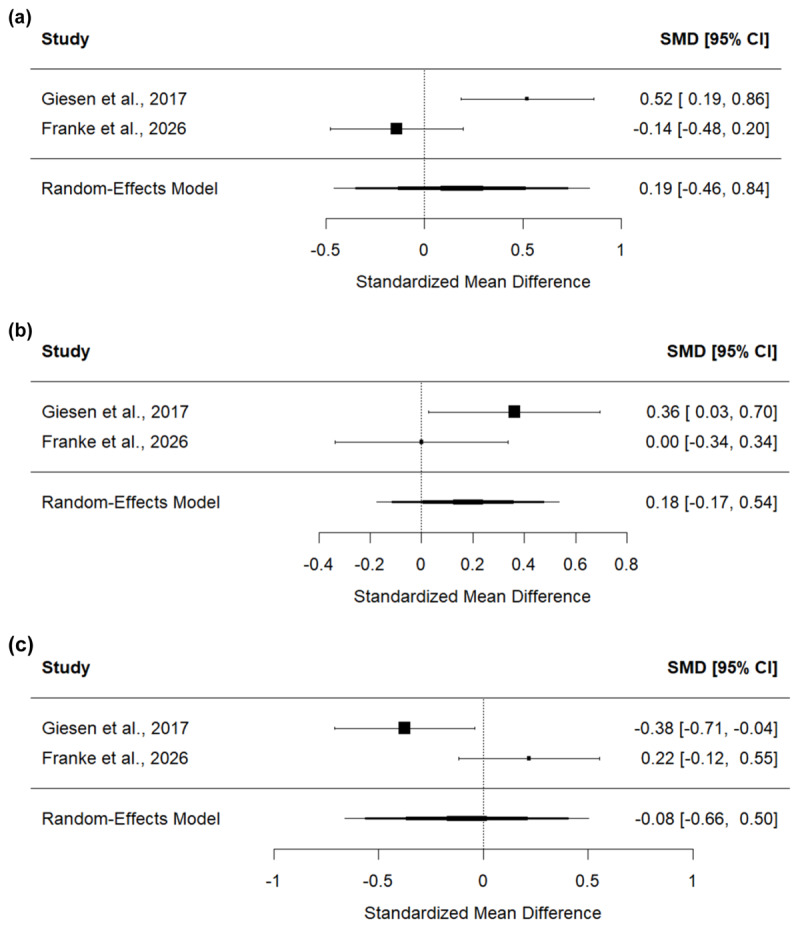
Forest plots for the error rates meta-analyses on **(a)** the modulating effect of vicarious feedback on oSRBR effects and **(b)** the occurrence of oSRBR effects after positive and **(c)** negative feedback respectively.

In our study, oSRBR effects were not affected by vicarious prime feedback. This clearly deviates from the findings of Giesen et al. (2017). Thus, to get a more robust estimation of the modulatory influence of vicarious feedback on oSRBR effects with more statistical power, we conducted a mini meta-analysis of both experiments (i.e., Giesen et al., 2017 and our replication). The random-effects meta-analysis was conducted in R with the *metafor* package (Viechtbauer, 2010) on standardized effect sizes *d_z_* of the contrast between interaction effect scores after positive vs. negative feedback of each experiment. Further, the meta-analysis was conducted separately for both error rates and RTs. Results revealed a mean effect size of *d_z_* = 0.13, 95% CI (–0.24, 0.49) for RTs (see Figure 3a) and a mean effect size of *d_z_* = 0.19, 95% CI (–0.46, 0.84) for error rates (see Figure 4a). This implies that there was no significant modulatory effect of vicarious feedback on oSRBR effects.

Further, we tested the occurrence of oSRBR effects separately for each vicarious prime feedback level. To this end, for each feedback condition (positive and negative) and dependent variable (error rates and RTs), we conducted a random-effects meta-analysis on standardized effect sizes *d_z_* of the test of interaction effect scores against zero. The analysis revealed that no siginicant standard oSRBR effects emerged across the studies, neither after positive (RTs: *d_z_* = 0.18, CI[–0.27, 0.64], see Figure 3b; error rates: *d_z_* = 0.18, CI[–0.17, 0.54], see Figure 4b), nor after negative feedback (RTs: *d_z_* = 0.05, CI[–0.19, 0.28], see Figure 3c; error rates: *d_z_* = –0.08, CI[–0.66, 0.50], see Figure 4c). Taken together, the results of the meta-analysis do not support our original hypothesis that vicarious feedback is used to make goal-inferences or any form of modulatory influence of vicarious feedback on observational SR binding in general.

## General Discussion

The present experiment was an exact replication of the study by Giesen et al. (2017). We conducted this replication in the light of recent literature finding that affective consequences and feedback generally do not affect binding and retrieval processes. Thus, we wanted to see whether the influence of vicarious feedback on oSRBR effects is robust and may therefore represent a special case, or if it is more likely that the results of Giesen et al. (2017) were a chance finding. First, we found main effects for vicarious prime feedback in RTs, with responses being faster after positive feedback compared to negative feedback. This implies that participants processed and were affected by our feedback manipulation. Second, we found that significant standard oSRBR effects only occurred after positive feedback, while oSRBR effects were absent after negative feedback. However, this difference between feedback conditions was not significant. Therefore, overall, our results suggest that binding and retrieval by observation was not affected by vicarious prime feedback. This clearly deviates from the findings of Giesen et al. (2017), as retrieval effects were not reversed after negative feedback, but absent. Because of this, we conducted a meta-analysis to get a more robust estimation of the modulatory influence of vicarious feedback on binding and retrieval by observation with the combined data of both experiments that have investigated this topic. The meta-analysis revealed no significant effects, suggesting that vicarious feedback does not modulate binding and retrieval by observation.

Taken together, our results neither support a modulatory influence of vicarious feedback on oSRBR effects in general nor the idea that SR bindings may represent propositional information. The latter finding is in line with other studies that directly or indirectly tested whether SR binding and retrieval effects code propositional inferences. For instance, in a conceptually similar study, Foerster et al. (2024) investigated the influence of positive vs. negative feedback on binding and retrieval after guessing trials, that is, in trials in which participants had no response rule but had to guess the correct response to a stimulus. Significant SR binding and retrieval effects only emerged after positive feedback but not after negative feedback. As their task only used two response options, a logical deduction of the correct response was possible. However, no binding and retrieval of inferred correct responses occurred after negative feedback, thus implying that propositional information were not integrated into SR bindings.

Furthermore, our results support and expand on previous findings on the influence of feedback on SR binding and retrieval processes. In line with several recent studies (Martini et al., 2025; Mocke et al., 2025; Parmar & Rothermund, 2024; Schöpper et al., in press), we found that oSRBR effects did not differ between feedback conditions. This is not trivial, as in our study, feedback had a different meaning than in those previous studies: In past research, the focus was typically on the affective consequences associated with the feedback. However, feedback did not signal whether or not a response was actually correct. This would have been hard to manipulate, as previous studies investigated retrieval of SR bindings formed from self-performed actions (i.e., one participant responded in every trial). Thus, participants had good insight into whether their responses were correct or not, which makes it impossible to manipulate feedback independently. The focus on binding and retrieval of observed responses in our studies allowed us to address this gap: As participants were not able to see the target (i.e., the font color) during their interaction partner’s response, we could use feedback to suggest whether or not a response was correct. Therefore, the fact that we still found that the size of oSRBR effects did not differ between feedback conditions strengthens the claim that external feedback has no modulatory influence on SR binding and retrieval effects.

While our findings match with the literature in suggesting that SR bindings do not represent propositional information and that SR binding and retrieval is not modulated by feedback, they can still appear surprising from the perspective of research on action slips. Here it has been well established that committing an error leads to goal-based binding and retrieval of the correct response (Foerster et al., 2023; Foerster et al., 2022; Foerster et al., 2021; Parmar et al., 2022). It remains unclear why the same does not happen if vicarious feedback signals that an observed response was incorrect. This discrepancy may be explained by focusing on the differences between the two scenarios of committing an error and observing a response that receives negative feedback. In a typical paradigm used to investigate binding and retrieval, people should generally have good insight on whether their response was correct or not. That is, because response rules are typically simple and participants can see the task-relevant target feature in every trial. Thus, when responding incorrectly, participants likely become aware of this quickly, potentially already during the execution of the erroneous response. This idea is supported by findings that show that explicit feedback is not necessary for binding of the correct response to occur after incorrect responding (Foerster et al., 2023). Further, theories on error processing suggest that that the correct action is represented quite strongly during error commission (Crump & Logan, 2013). Therefore, the correct response may already be activated during responding, which may facilitate its integration into the SR binding. In contrast, in the interactive color classification task that we used, responses to stimuli are first observed and feedback is only presented after response execution. Therefore, observers can only realize that the observed response may have been incorrect at a much later point during the binding process. It is possible that this may be too late to still integrate the alternative response into the SR binding.

Note, however, that the previous two points are somewhat limited by the experiment’s design, because negative feedback could signal either that a response was correct but too slow, or that it was incorrect. Thus, we do not know for sure whether participants interpreted negative feedback as indicative of an error. Consequently, the present experiment is not an unambiguous test for propositions. We addressed this issue in an additional experiment that distinguishes between negative feedback due to slow, but correct vs. incorrect responding (see Supplementary Material for details). However, findings of this additional experiment essentially mirrored those of the present experiment: That is, oSRBR effects were unaffected by vicarious feedback, therefore strengthening our argument.

It is also noteworthy that our findings do differ from previous studies on the influence of feedback on SR binding in one important way: Typically, it is found that standard binding and retrieval effects emerge in all feedback conditions. In contrast, we found only small or no oSRBR effects at all. The absence of significant general oSRBR effects in the meta-analysis somewhat limits the interpretability of our results, as one could say that there were no effects to be modulated by feedback in the first place. Further, this finding suggests that the introduction of vicarious feedback did have some effect on oSRBR effects in general. That is, because the very same paradigm without vicarious feedback typically produces robust oSRBR effects, particularly with the inclusion of a positive interdependence manipulation (cf. Giesen et al., 2014). A possible reason why the presence of vicarious feedback may have prevented the emergence of oSRBR effects is that may have led participants to regard the observed responses as less relevant. This reasoning is motivated by findings showing that social relevance modulates oSRBR effects (Giesen et al., 2014; Giesen et al., 2018). Due to the relatively high proportion of negative feedback that interaction partners and participants themselves received, they may have perceived the performance of their partner as too unreliable to use their responses to guide their own performance. In other words, they may have perceived their interaction partner as not particularly competent in the context of this particular task. This may lead them to pay less attention to their responses, resulting in features of stimulus and observed response receiving less activation, which would reduce the likelihood of them being integrated into an observationally acquired SR binding and retrieved later on (cf. Giesen, 2024).

## Conclusion

Our findings suggest that vicarious feedback does not modulate oSRBR effects, even if feedback indicates whether or not an observed response was correct. This is in line with recent research suggesting that SR binding and retrieval is an automatic process that is impenetrable by affective consequences like feedback. We could not replicate the findings of Giesen et al. (2017), as oSRBR effects were not reversed after negative feedback. This implies that no inferred action goals were bound. Therefore, the present results do not support the idea that SR bindings represent propositional information.

## Data Accessibility Statement

All data, analysis scripts, and experimental files are available on this paper’s project page on the Open Science Framework (https://osf.io/rntf9).

## Additional File

The additional file for this article can be found as follows:

10.5334/joc.494.s1 Supplementary Material. Additional experiment.

## Footnotes

As preregistered, we also calculated Balanced Integration Scores (BIS) to analyze a composite score that channels systematic variance from RT and error rates to the same dependent variable. BIS were computed according to Liesefeld and Janczyk (2019) as *z*_PC_ – *z*_RT_, with percentage correct (PC) being 1-ERR. However, the analysis of BIS revealed no significant effects of theoretical interest and is therefore not reported in detail.
