# Supplementary material for: Negative Feedback Does Not Reverse Observationally Acquired Binding and Retrieval Effects: A Failed Replication
Source: J Cogn. 2026 Mar 27;9(1):22. doi: 10.5334/joc.494 (PMC13025250; doi:10.5334/joc.494)
Supplement: Supplementary Material. — Additional experiment. [file joc-9-1-494-s1.pdf]

Supplementary material

With our replication of the study by Giesen et al. (2017) we demonstrated that observationally acquired stimulus-response binding and retrieval (oSRBR) effects are not affected by vicarious feedback and likely do represent propositional information. However, one could still argue that the design of the study is not ideal to investigate the role of propositions in observational SR binding. That is because negative feedback did not always signal an error but could also indicate that a response was correct but too slow. Whether or not propositions influence behavior, in this case oSRBR effects, depends on the extent to which propositions are considered to be true (De Houwer, 2009). Thus, valid inferences of action goals based on observed responses and vicarious feedback would only be possible if the negative feedback signaled that an error was committed.

Accordingly, in this supplementary experiment, we aimed to further examine the influence of vicarious feedback on oSRBR effects by clearly dissociating feedback for incorrect responses (error feedback) from feedback that indicates correct but slow responses (time-based feedback). This separation also allows for an unambiguous test of whether observational SR bindings represent propositional information. To investigate this, we modified the design by Giesen et al. (2017). Participants performed the same dyadic color categorization task in pairs with each response being followed by feedback. However, we used three clearly distinguishable types of feedback. *Positive feedback* indicated that a response was both correct and fast. There were two types of negative feedback: The first type signaled to participants that a response was correct but too slow (this feedback type will further be referred to as *time-based feedback*) while the other negative feedback type indicated erroneous responding (further referred to as *negative feedback*). If observationally acquired SR bindings are indeed propositional and represent inferred action goals, then standard oSRBR effects should emerge after positive and time-based feedback because these feedback types indicate that the observed response was correct. In turn, *reversed* oSRBR effects should only emerge after negative feedback because it indicates an error and therefore allows for an inference on correct action goals.

Method

Transparency and openness

Prior to data collection, the exact method, design, data preparation, and planned analyses were preregistered online at the Open Science Framework (OSF, https://osf.io/xcb4n). All data, analysis scripts, and experimental files will be made available after initial acceptance of the paper (link for review: https://osf.io/rntf9/?view_only=b3f4a391fa8e452ca71c869fe38f6795). The experiment was in accordance with the Ethical standards of the Institute of Psychology of the University of Jena and the Declaration of Helsinki.

Participants

In a pilot study, an effect of $d_z = 0.29$ was observed for the contrast between the positive vs. negative prime feedback conditions. According to an a priori power analysis with G*Power 3.1.9.7 (Faul et al., 2007), a sample size of n=75 was necessary to detect an effect of $d_z = 0.29$ with a power (1-ß) = 0.80 in a one-tailed *t*-test for dependent samples (contrasting oSRBR effects after positive vs. negative feedback) with $\alpha=0.05$.

Overall, 78 students of the Friedrich-Schiller University of Jena participated in the experiment. All participants were native German speakers. Participants were tested in pairs and received 9€ and chocolate as an extra reward. Based on the written protocol of the experimenter, one participant had to be excluded because they second-guessed the feedback manipulation. Another 7 participants were excluded because of excessive error rates (>25%) in the color categorization task or in the memory test. Thus, data of 70 participants (38 male, 31 female, 1 non-binary; $M_{age} = 23$, $SD_{age} = 5.8$) were analyzed.

Apparatus and stimuli

The experiment was programmed with E-Prime 3.0. Participant pairs were placed opposite of each other, each in front of a 21-inch screen. The screens were positioned to prevent eye contact between participants (Figure S1a). However, they could still see the two

response pads that were used to collect responses, and thus the other participant's responses, with their peripheral vision. One of these response pads had a green and the other one a yellow push button in the middle. Both had two black rest state-keys in front of and behind the push button. During the color categorization task, participants continuously pressed the two rest-state keys on their side with their right and left hand and only released those keys to respond by hitting the yellow or the green button. The response pads were connected to the computer via a parallel port.

We used 25 neutral mono- or disyllabic German adjectives as stimuli (e.g., glatt, eben, kurz; in English: smooth, even, short). These adjectives were presented centrally on each participant's screen in Times New Roman font (font size: 16 pt). They were either presented in white (RGB: 255, 255, 255), green (RGB: 20, 156, 23), yellow (RGB: 255, 255, 102), or yellow-green (RGB: 216, 255, 102) color. For auditory feedback, we used three computer-generated sounds, presented to participants via headphones. For positive and negative feedback, sounds started at 440 Hz. Positive feedback sounds increased in steps of 20 Hz (each step lasting for 15 ms) until a frequency of 840 Hz was reached. For negative feedback, sounds decreased at the same rate until a frequency of 40 Hz was reached. This resulted in sounds that are intrinsically positive (increasing frequencies) or negative (decreasing frequencies; cf. Rothermund, 2003). Total sound duration of the positive and the negative feedback sound was 300 ms. In the case of time-based feedback, a shorter sound (75ms) with a constant frequency of 75 Hz was played.

Procedure

First, demographic information (age, gender, study field) was collected, and participants gave their informed consent to participate; otherwise the study was terminated. Then written instructions for both participants were presented on each of their screens. Participants were informed that they would perform a color categorization task together with the other participant. This task followed a socially shared prime-probe design and was used to assess oSRBR effects. In both the prime and the probe trial the participants' task was to

categorize the font color of the presented word stimulus as fast and accurately as possible by pressing the corresponding (i.e., yellow/green) push button in the middle of the response pads. In contrast to previous studies on oSRBR effects we used yellow and green as target colors, as these are more similar colors and thus harder to distinguish than red and green. This was done to make a high error rate and therefore a frequent occurrence of negative feedback more plausible for participants. Word stimuli could appear with an equal likelihood of 33% in either yellow, green, or yellow-green font. The inclusion of yellow-green stimuli was the second means to increase the face validity of our feedback manipulation for participants. Importantly, participants were made to believe that all words would clearly be identifiable as either yellow or green and that therefore there would always be one unambiguously correct response. However, there was no correct response in trials with yellow-green stimuli, therefore they are considered *unsolvable trials*, while trials with green or yellow words are considered *solvable trials*. Further, participants were told that after each response they would both receive feedback depending on whether the actor’s response was fast and correct (positive feedback), correct but slow (time-based feedback), or incorrect (negative feedback). However, feedback was only authentic in solvable trials. In unsolvable trials it was determined by a different mechanism to increase the frequency of negative feedback (see Design for details).

In each prime or probe trial, only one participant (the actor in the respective trial) saw the word in green, yellow, or yellow-green and performed the color categorization response. The other participant (the observer) saw the same word in white font and had to observe the actor’s response. Participants responded in strict alternation: During the first half of the experiment (prime-probe sequences 1-180), participant A was the prime actor/probe observer, meaning that participant A always gave the color categorization response in the prime trial and observed participant B’s response in the probe trial. Participant B was the prime observer/probe actor. For the second half of the experiment (prime-probe sequences 181-360) these roles were switched: now participant A was prime observer/probe actor and participant B prime actor/probe observer.

Each prime-probe sequence of the color categorization task (see Figure S1b) began with a ready signal ("!!!") that was presented centrally on both participants' screens in white font against a black background for 500 ms, followed by a fixation cross (250 ms). Next, the prime word was presented until the prime actor responded or until 1500 ms had passed (i.e., the prime actor failed to respond in time). The word was shown in yellow, green, or yellow-green font to the prime actor and in white font to the prime observer. Then, feedback for the prime actor's response was shown to both participants for 500ms. In case of positive feedback, participants saw a smiley face and heard the positive feedback sound. Time-based feedback led to the presentation of an hourglass and the time-based feedback sound. Negative feedback included a schematic grumpy face and the negative feedback sound. After that, the probe trial began with the presentation of another fixation cross for a variable duration of 150 to 350 ms (M = 250 ms). Then the probe word (printed in green, yellow, or yellow-green for the probe actor and in white for the probe observer) appeared on the screen until the probe actor responded or until a maximum of 1500 ms elapsed. Both participants also received feedback for the probe actor's response (duration: 500 ms). This probe feedback was determined by the same rules as the prime feedback. However, probe feedback was not of theoretical interest and was only used to make conditions comparable for both prime and probe actors.

To ensure that prime observers attended to prime responses, 25% of all probe trials were followed by a memory test. Prime observers were asked to repeat the prime actor's response they had just observed by pressing the corresponding push-button (until response). Each prime-probe sequence ended with a blank black screen (1250 ms).

Before the start of the first block of the main experiment, participants completed a practice block consisting of 18 prime-probe sequences. The practice block had to be repeated if more than 20% of the categorization responses were incorrect. If participants did not pass the practice block upon the third try the experiment was terminated. Additionally, there was a shorter practice block of 8 prime-probe sequences before the second block of the main experiment for participants to get accustomed to the role change. Similar to Giesen

et al. (2017), to increase the likelihood of the occurrence of oSRBR effects, we adopted the "positive interdependency" manipulation that was first used by Giesen et al. (2014). Before the start of the main block, participants were instructed to work together and were told that they would only receive an extra reward (a chocolate bar) if they both performed well in terms of accuracy (i.e., less than 25% errors in the color categorization task and in the memory test). Participants then worked through two experimental blocks of 180 prime-probe sequences each. After every 60 prime-probe sequences, participants could take a short break. During these breaks, the interdependency manipulation was refreshed by reminding participants to work together to obtain the extra reward.

After completion of the color categorization task, participants were asked to fill out a questionnaire on how they experienced the experimental situation and their interaction partner. On 7-point Likert scales they rated the situation as cooperative (1) vs. competitive (7; one item) and comfortable vs. uncomfortable (averaged across three items: 1 = difficult/unpleasant/negative; 7 = easy/pleasant/positive; Cronbach's α = .80). Further, they were asked how agreeable they perceived their interaction partner (averaged across four items: 1 = disagreeable/insecure/unfriendly/incompetent; 7 = agreeable/confident/friendly/competent; Cronbach's α = .52). Finally, participants were debriefed and rewarded.

Design

The study comprised a 2x2x3 within-subjects design with the factors stimulus relation, response relation, and vicarious prime feedback. Stimulus relation was manipulated by either repeating or changing the word stimulus from prime to probe (50% stimulus repetitions, 50% stimulus changes). Response relation was manipulated by having probe actors either perform the same response they had previously observed in the prime (response repetition; e.g., yellow-yellow) or a different response (response change; e.g., green-yellow)[i]. After each prime response either positive, time-based, or negative feedback was audio-visually presented to both participants. Participants were told that positive feedback indicated that a

response was both correct and fast; time-based feedback meant that the response was correct but too slow; negative feedback meant that the response was incorrect. This was true for solvable prime trials (i.e., stimulus presented in green or yellow), but not for unsolvable prime trials (i.e., stimulus presented in yellow-green, therefore no correct response was possible).

In solvable trials, participants received negative feedback for incorrect responses. After correct prime responses, positive feedback was presented if the response was faster than the median of the RT-distribution of the 14 preceding correct prime and probe responses (moving median technique, cf. Giesen et al., 2017). Time-based feedback was presented when the response was slower than the moving median. In unsolvable prime trials, correct responding was not possible. Therefore, feedback was solely determined by the rank of the current prime response's RT compared to the RT-distribution of 13 preceding unsolvable prime and probe trials: If the current RT was faster than the fourth-ranking RT of the preceding trials or equal or slower than the tenth ranking RT, negative feedback was given. For RTs slower than the fourth but faster than the tenth-ranking RT, feedback type was randomly determined with equal probability for positive, time-based, and negative feedback. Overall, this resulted in rates of 41% positive, 33% time-based, and 26% negative prime feedback. Note that participants also received feedback after probe responses and that this feedback was determined by the same rules as prime feedback. However, probe feedback was not of theoretical interest in this study. Similar to prime trials, probe trials were either solvable or unsolvable (following the same rules as for prime trials). Like Giesen et al. (2017), error rates (ERR) in probe trials and probe release RTs of rest-state keys in solvable probe trials served as our main dependent variables of interest.[ii]

Data preparation

We only used prime-probe sequences with solvable probe trials for all analyses testing our main hypothesis (66.7% of all prime-probe sequences). Probe trials with errors in the memory test (5.89%, 1.96% of all solvable probe trials) and probe responses faster than

300ms or slower than 3 interquartile ranges above the 75th percentile of the individual RT distribution (Tukey, 1977) were discarded prior to all analyses (1.13%). For probe RT analyses, we also excluded trials with erroneous probe responses (3.5%). Deviating from our preregistration, we also excluded trials in which the probe actor accidentally responded in the prime trial or vice versa (2.12%), as the resulting SR binding would be based on a self-performed and not on an observed response. Means for probe RTs and ERR for all conditions of the factorial design are provided in Table S1. We computed effect scores for oSRBR effects representing the stimulus relation x response relation interaction for each participant in each vicarious prime feedback condition. This score is calculated by subtracting the performance costs of stimulus repetitions compared to stimulus changes in response change trials from the performance benefit of stimulus repetitions vs. changes in response repetition trials (see Table S1 for computation). Standard oSRBR effects are reflected by effect scores significantly larger than zero, while reversed oSRBR effects would be indicated by effect scores smaller than zero.

Results

Statistical analyses were performed with R (R Core Team, 2022; Version 4.1.2). Bayes Factors were computed with JASP (Version .14.1.0).

Manipulation checks

A *t*-test against the scale midpoint (4) using *t*-tests revealed that participants perceived the situation as cooperative, M = 3.41, $|t|(69)$ = 3.15, p = .002, $|d|$ = 0.38, indicating that positive interdependence was successfully induced. Further, the situation was experienced as neither particularly comfortable nor uncomfortable, M = 3.91, SD = 0.93. The interaction partner was judged as agreeable, M = 5.37, SD = 1.00.

Error rates in the memory test were compared as a function of vicarious prime feedback in a one factorial ANOVA. This was done to assess whether attention for observed responses differed depending on prime feedback, which could affect retrieval of observational SR bindings. However, this was not the case, as memory errors did not differ

systematically $F(2, 138) = 0.61$, $p = .499$, $\eta_p^2 = .01$ ($M_{positive} = 2.76\%$, $M_{time\text{-}based} = 3.01\%$, $M_{negative} = 3.72\%$).

Preregistered analyses

Response times

We conducted a 2 (stimulus relation: stimulus repetition vs. change) x 2 (response relation: response repetition vs. response change) x 3 (vicarious prime feedback: positive vs. time-based vs. negative) repeated measures analysis of variance (ANOVA) on mean probe RTs to analyze probe performance. Global ANOVA results are presented in Table S2. The ANOVA showed that responses were faster when word stimuli repeated from prime to probe ($M = 502$ ms) than when the word changed ($M = 506$ ms). There was also a main effect of feedback, with responses being faster after positive feedback ($M_{positive} = 500$ ms, $M_{time\text{-}based} = 504$ ms, $M_{negative} = 507$ ms). Further, the analysis revealed a significant interaction of stimulus relation and response relation, indicating retrieval of observationally acquired SR bindings. However, the three-way interaction was not significant ($BF_{01} = 19.50$), meaning that oSRBR effects did not differ depending on vicarious feedback (see Figure S2). All other effects were not significant ($F \leq 3.84$, $p \geq .053$).

Despite of finding a significant oSRBR effect overall, *t*-tests of interaction effect scores against 0 were not significant in any prime feedback condition ($SxR_{positive} = 6$ ms, $t(69) = 1.09$, $p = .279$, $d = 0.13$, $BF_{01} = 4.31$; $SxR_{time\text{-}based} = 8$ ms, $t(69) = 1.48$, $p = .143$, $d = 0.18$, $BF_{01} = 2.69$; $SxR_{negative} = 7$ ms, $t(69) = 0.90$, $p = .369$, $d = 0.11$, $BF_{01} = 5.14$).

Error rates

The 2 (stimulus relation) x 2 (response relation) x 3 (vicarious prime feedback) ANOVA on probe actors' mean error rates (see Table S2 for global ANOVA results) revealed no significant effects ($F \leq 2.94$, $p \geq .056$). Importantly, this included both the two-way interaction of stimulus relation and response relation ($F < 1$) and three-way interaction ($F < 1$,

BF_{01} = 12.36), indicating the absence of oSRBR effects and of any modulation of those effects by vicarious prime feedback in error data.

Following the preregistered analyses, we also tested S x R interaction effect scores against zero for each vicarious prime feedback condition. None of the *t*-tests were significant ($SxR_{positive}$= -0.24%,: |*t*|(69) = 1.41, *p* = .163, |*d*| = .17, BF_{01} = 2.96; $SxR_{time\text{-}based}$= -0.39%, |*t*|(69) = 0.45, *p* = .651, |*d*| = .05, BF_{01} = 6.89; $SxR_{negative}$= -0.18%, |*t*|(69) = 0.17, *p* = .869, |*d*| = .02, BF_{01} = 7.52), suggesting that no oSRBR effects emerged in any of the conditions in the error data.

Exploratory analyses: Response repetition rates in unsolvable probe trials

For an additional exploratory test for retrieval of observationally acquired SR bindings, we calculated response repetition rates in unsolvable probe trials for all combinations of stimulus relation and vicarious prime feedback levels (see Table S3 for means). Response repetition rates reflect the percentage of unsolvable probe trials in which a probe actor repeated the response they had previously observed in the prime. We expected a higher rate of response repetitions for stimulus repetitions vs. changes after positive and time-based vicarious prime feedback. After negative feedback, we expected a smaller (reduced, absent or reversed) effect of stimulus repetitions vs. changes on response repetition rates. This was tested by entering response repetition rates into a 2 (stimulus relation: stimulus repetition vs. stimulus change) x 3 (vicarious prime feedback: positive vs. time-based vs. negative) ANOVA. There was main effect of stimulus relation, $F(1, 69) = 4.19$, $p = .044$, $\eta_p^2 = .06$, suggesting that participants repeated previously observed responses more frequently when word stimuli repeated from prime to probe (M = 50.7%) than when they changed (M = 47.4%). However, neither the main effect of prime feedback, $F(2, 138) = 0.88$, $p = .416$, η_p^2 = .01, nor the interaction of stimulus relation and prime feedback were significant, $F(2, 138)$ = 0.29, $p = .750$, $\eta_p^2 < .01$, indicating that vicarious prime feedback did not influence response repetition rates in any way.

Discussion

We investigated whether vicarious prime feedback modulates retrieval of observationally acquired SR bindings when negative feedback due to errors is dissociable from negative feedback due to slow (but correct) responses. We expected reversed oSRBR effects after negative feedback due to errors and standard oSRBR effects after positive and time-based feedback. Vicarious prime feedback influenced performance in general, as indicated by the main effects of feedback on RT. This implies that the feedback was processed by participants and affected them during the color categorization task. Further, we found a small but significant standard oSRBR effect in RTs. Contrary to our expectations but in line with our main experiment, the oSRBR effect was not modulated by vicarious prime feedback. This suggests that participants simply bound the observed response independently of the feedback that was presented to them and did not actively infer action goals. Thus, our findings do not support the claim that SR bindings represent propositional information, nor does it support the claim that feedback has a reinforcing (affect-based) general effect on oSRBR effects. This contradicts the findings of Giesen et al. (2017).

Overall, the findings of this experiment support the idea that vicarious feedback in fact does not influence oSRBR effects, similar to a lack of feedback effects on standard SRBR effects (Martini et al., 2025; Mocke et al., 2025; Parmar & Rothermund, 2024; Schöpper et al., in press). Apart from the study by Giesen et al. (2017), evidence for goal-based binding has only been found in the context of action slips. Committing an error yourself, however, is different from receiving negative feedback for an observed action and then concluding that the observed action must have been incorrect and that another action should have been executed instead.

It must also be mentioned that there is at least one alternative explanation for our findings in this experiment that concerns differences in the experimental designs compared to Giesen et al. (2017). Our experiment was more complex for participants, as we used three different types of feedback instead of two. Further, we also introduced unsolvable trials, which could have caused confusion in some participants. This latter idea is supported by several comments from participants in the post-experimental questionnaire. Thus, the higher

complexity of our task may have led to participants feeling overwhelmed, thus potentially discrediting the feedback they were presented with. However, in light of the finding in our main experiment and recent literature in general, it appears more likely that oSRBR effects are simply not modulated by vicarious feedback.

[ii] Note that an experimental manipulation of response relation was only possible in prime-probe sequences with a solvable prime and a solvable probe trial (i.e., if the word is not presented in yellow-green) since there is no correct response in unsolvable trials. Because of that, sequences with unsolvable probe trials were excluded from our main analyses (see also Data Preparation).

[ii] We also calculated Balanced Integration Scores (BIS) to analyze a composite score that channels systematic variance from RT and error rates to the same dependent variable. BIS were computed according to Liesefeld and Janczyk (2019) as z_{PC} - z_{RT}, with percentage correct (PC) being 1-ERR. However, the analysis of BIS revealed no significant effects of theoretical interest and is therefore not reported in detail.

Tables

Table S1

Probe performance M (SD) in the observational SR binding paradigm

		% errors		RT (ms)	
Vicarious prime feedback		RR	RC	RR	RC
Positive	Stimulus repetition (SR)	0.70 (2.92)	1.27 (3.34)	494 (65)	503 (64)
	Stimulus change (SC)	1.64 (4.97)	1.23 (4.04)	500 (64)	504 (74)
	Δ SC - SR	0.94 [0.5]	-0.04 [0.6]	6* [3.1]	1 [4.2]
	S x R interaction score	0.98 [0.7]		5 [5.1]	
Time-based	SR	2.64 (5.99)	1.78 (4.16)	497 (72)	507 (72)
	SC	2.31 (6.07)	1.96 (5.42)	505 (77)	507 (74)
	Δ SC - SR	-0.33 [0.8]	0.18 [0.7]	8* [3.8]	0 [3.8]
	S x R interaction score	-0.51 [1.1]		8 [5.4]	
Negative	SR	1.62 (5.11)	1.85 (5.66)	503 (69)	509 (77)
	SC	1.31 (4.19)	1.76 (5.12)	509 (68)	508 (74)
	Δ SC - SR	-0.31 [0.7]	-0.09 [0.9]	6 [5.1]	-1 [5.5]
	S x R interaction score	-0.22 [1.3]		7 [7.3]	

Note. RR = response repetition, RC= response change. S x R interaction score = $(\Delta\ SC - SR)_{RR}$ - $(\Delta\ SC - SR)_{RC}$. Standard error of the mean in brackets. * $p < .05$. ** $p < .01$. *** $p < .001$. Asterisks denote that effects significantly differ from zero

Table S2

ANOVA results on probe actors' mean error rates and mean RTs

	Variables	*df*1	*df*2	*F*	*p*	η_p^2
Errors						
	Stimulus relation (S)	1	69	0.03	.903	<.01
	Response relation (R)	1	69	0.03	.864	<.01
	Prime Feedback (F)	2	138	2.94	.056	.04
	S x R	1	69	0.02	.901	<.01
	S x F	2	138	0.55	.562	.01
	R x F	2	138	0.86	.424	.01
	S x R x F	2	138	0.64	.508	.01
RT						
	S	1	69	5.04*	.028	.07
	R	1	69	3.84	.053	.05
	F	2	138	3.91*	.022	.05
	S x R	1	69	4.24*	.043	.06
	S x F	2	138	0.04	.945	<.01
	R x F	2	138	0.32	.727	<.01
	S x R x F	2	138	0.04	.943	<.01

Note. * $p < .05$. ** $p < .01$. *** $p < .001$.

Table S3

Mean response repetition rates (%) in unsolvable probe trials

Vicarious prime feedback	Stimulus repetition	Stimulus change
Positive	52.2	49.2
Time-based	49.9	47.9
Negative	50.1	45.1

Figures

Figure S1

Schematic illustration of (a) the experimental setup and (b) and a sample trial sequence from the perspective of each co-actor

(a)

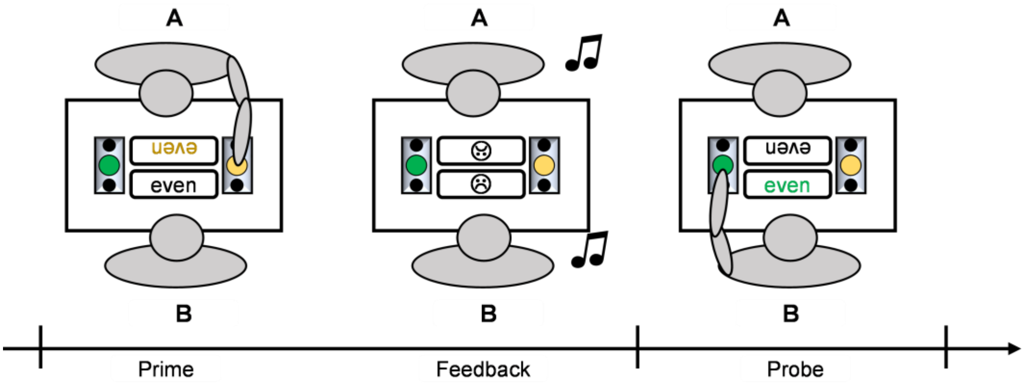

(b)

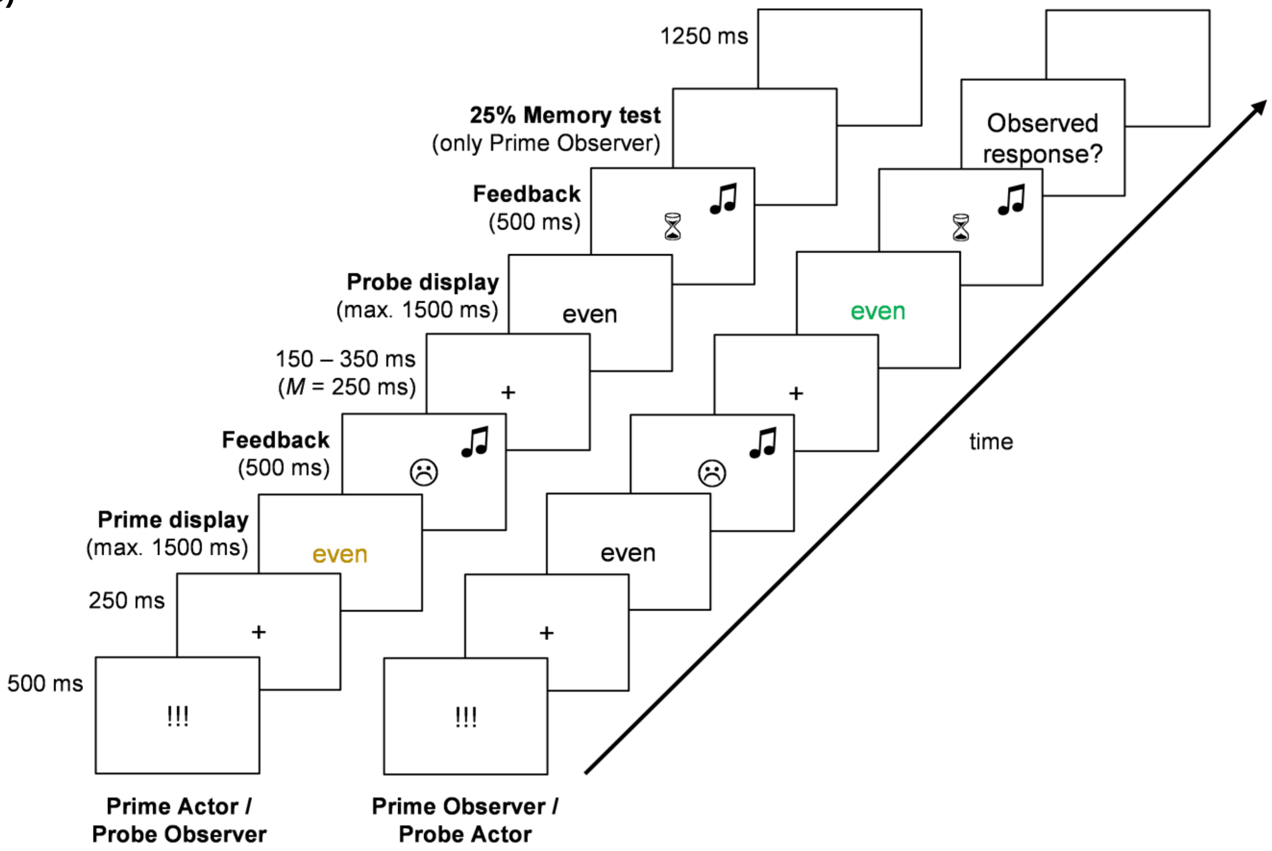

Note. Stimuli are not drawn to scale. For illustrative purposes, foreground and background colors are inverted. Font colors in the figure deviate slightly from font colors used in the experiment for better readability.

Figure S2

Probe performance (RT) as a function of stimulus relation, response relation, and vicarious prime feedback

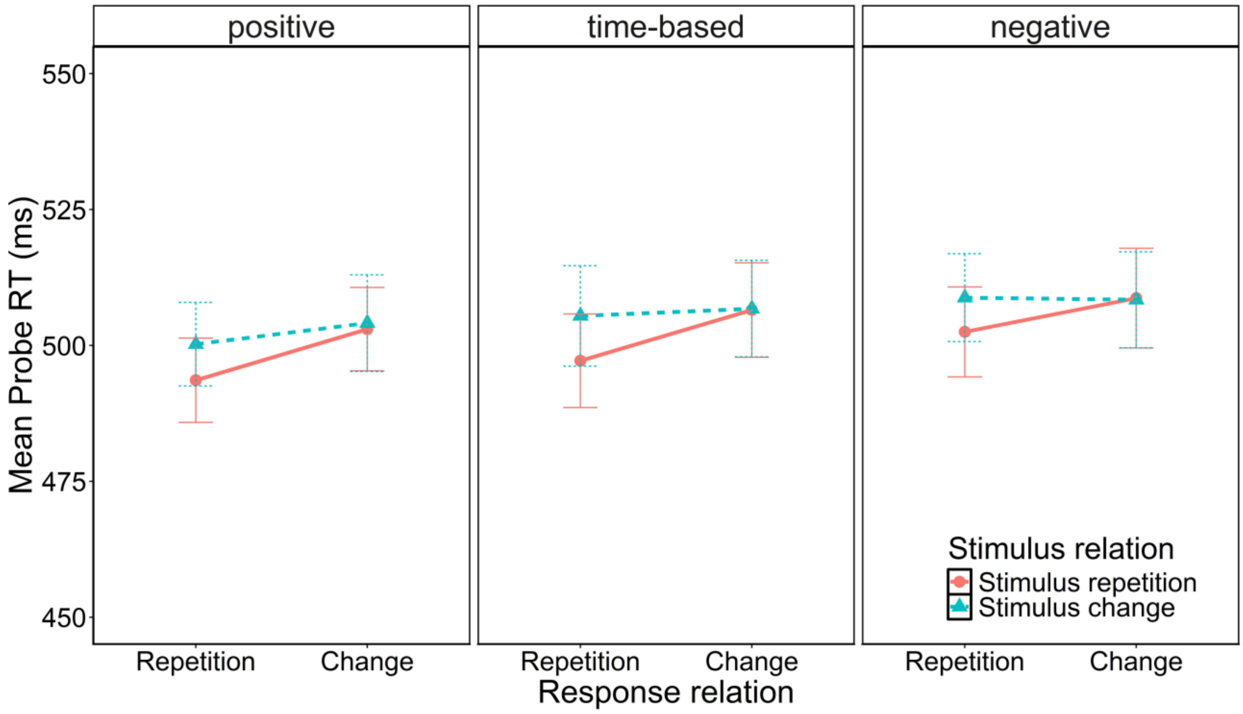

References

De Houwer, J. (2009). The propositional approach to associative learning as an alternative for association formation models. *Learning & Behavior*, *37*(1), 1–20. https://doi.org/10.3758/LB.37.1.1

Faul, F., Erdfelder, E., Lang, A.-G., & Buchner, A. (2007). G*Power 3: A flexible statistical power analysis program for the social, behavioral, and biomedical sciences. *Behavior Research Methods*, *39*(2), 175–191. https://doi.org/10.3758/BF03193146

Giesen, C., Herrmann, J., & Rothermund, K. (2014). Copying competitors? Interdependency modulates stimulus-based retrieval of observed responses. *Journal of Experimental Psychology: Human Perception and Performance*, *40*(5), 1978–1991. https://doi.org/10.1037/a0037614

Giesen, C., Scherdin, K., & Rothermund, K. (2017). Flexible goal imitation: Vicarious feedback influences stimulus-response binding by observation. *Learning & Behavior*, *45*(2), 147–156. https://doi.org/10.3758/s13420-016-0250-1

Martini, A., Rudolph, M., & Rothermund, K. (2025). Stimulus-response binding and retrieval is independent of affective consequences. *Manuscript Submitted for Publication*.

Mocke, V., Kunde, W., & Rothermund, K. (2025). Anticipated effect valence retrieves matching past responses but does not modulate stimulus-response binding. *Cognition and Emotion*, 1–17. https://doi.org/10.1080/02699931.2025.2566308

Parmar, J., & Rothermund, K. (2024). Nothing else matters: Stimulus-response binding and retrieval is independent of affective consequences. *Journal of Experimental Psychology. Learning, Memory, and Cognition*, *50*(3), 362–382. https://doi.org/10.1037/xlm0001288

R Core Team. (2022). *R: A language and environment for statistical computing* [Computer software]. R Foundation for Statistical Computing. www.R-project.org

Rothermund, K. (2003). Motivation and attention: Incongruent effects of feedback on the processing of valence. *Emotion (Washington, D.C.)*, *3*(3), 223–238. https://doi.org/10.1037/1528-3542.3.3.223

Schöpper, L.-M., Ogiermann, L., Eder, A. B., Rothermund, K., & Frings, C. (in press). Re-visiting an affective modulation of binding and retrieval. *Cognition and Emotion*.

Tukey, J. W. (1977). *Exploratory data analysis*. Reading, MA.
